# Generation of gastirc insulin-secreting organoids from human stomach sample

**DOI:** 10.21203/rs.3.pex-2147/v1

**Published:** 2023-04-27

**Authors:** Xiaofeng Huang, Qiao Zhou

**Affiliations:** Weill Cornell Medicine

**Keywords:** human, stomach, stem cells, differentiation, gastric insulin-secreting, GINS organoids, beta cells, diabetes, cell therapy, regeneration

## Abstract

Stomach stem cells are accessible by biopsy and propagate robustly in culture, offering an invaluable resource for autologous cell therapies. Here we describe a detailed protocol to isolate, expand, engineer and differentiate human gastric stem cells (hGSCs) into pancreatic islet-like organoids containing abundant gastric insulin-secreting (GINS) cells that resemble beta-cells in molecular hallmarks and function. Sequential activation of the inducing factors NGN3 and PDX1-MAFA led hGSCs onto a novel differentiation path, including endocrine progenitor and GINS precursor, before adopting beta-cell identity, at efficiencies close to 70%. GINS organoids acquired glucose-stimulated insulin secretion in 10 days post differentiation.

## Introduction

Gut stem cells are highly proliferative and power the weekly self-renewal of the gut mucosal lining^1-3^. Harvested from biopsies, human gut stem cells can be propagated in culture as organoids or primary cell lines over many generations, providing abundant tissues for potential autologous transplantation therapies^4-6^. Gut stem cells produce gut-specific tissues, including hormone-secreting enteroendocrine cells (EECs). Rare insulin expressing EECs have been reported in fetal human small intestine^7^. Whether such cells secret insulin is unknown, but their presence suggests an intrinsic permissiveness for insulin production in the fetal if not postnatal intestine. Generating functional insulin-secreting cells has tremendous therapeutic value, offering treatments for insulin-dependent diabetes, including the autoimmune type 1 diabetes^8-13^. An attractive feature of using gut stem cells to make beta-cell mimics is the ease of establishing autologous organoids from biopsies, which can enable mass production and personalized therapies.

Previously, we reported that co-expression of the endocrine regulator NEUROG3 (also known as NGN3) and pancreatic beta-cell regulators PDX1 and MAFA could induce insulin-secreting cells from murine intestine and stomach^14^. Here, we provided a detailed step-by-step protocol to induce cultured hGSCs derived from human donors to differentiate into islet-like organoids at high efficiency, containing approximately 70% beta-like cells and other islet-like endocrine populations. The protocol contains 4 sections including (1) isolation of primary hGSCs from human stomach sample, (2) expansion and cryopreservation of hGSCs, (3) generation of *Ngn3ER*-hGSC line, and (4) generation of GINS organoids. GINS organoids generated from this protocol exhibited glucose responsiveness 10 days after induction.

## Reagents

### General reagents

Fibronectin (Sigma-Aldrich, F4759)

Matrigel (VWR, 47743-722)

DMSO (Sigma-Aldrich, 472301)

DPBS without calcium and magnesium (Thermo Fisher Scientific, 14190144)

BSA, fatty acid free (VWR, 10842-692)

4-Hydroxytamoxifen (4-OH-TAM, Sigma-Aldrich, H7904)

Polybrene (Millipore Sigma, TR-1003-G)

TrypLE (Thermo Fisher Scientific, 12604021)

Trypsin-EDTA (0.25%), phenol red (Thermo Fisher Scientific, 25200072)

Rabbit anti-MAFA (Bethyl; A700-067; 1:1000)

Opti-MEM I Reduced Serum Medium (Thermo Fisher Scientific, 31985062)

Sodium Pyruvate, 100 mM (Thermo Fisher Scientific, 11360070)

Lipofectamine 3000 Transfection Reagent (Thermo Fisher Scientific, L3000015)

Puromycin dihydrochloride (Sigma-Aldrich, P8833)

### Lentivirus

**Note:** lentiviral plasmids are available from Addgene under a uniform biological material transfer agreement. Virus can be packaged as previously decribed^15,16^ or following the protocol on the website (https://www.thermofisher.com/us/en/home/life-science/cell-culture/cell-culture-learning-center/cell-culture-resource-library/cell-culture-transfection-application-notes/improve-lentiviral-production-using-lipofectamine-3000-reagent.html).

Lenti-EF1α-*Ngn3ER-2A-PuroR-2A-mCherry* (EF1α, EF-1α promoter; 2A, 2A peptide for polycistronic gene expression; *PuroR*, puromycin resistant gene)

Lenti-CMV-*Pdx1-2A-Mafa* (CMV, CMV promoter; 2A, 2A peptide for polycistronic gene expression)

### Cell lines

DR4 MEF, irradiated (Thermo Fisher Scientific, A34966)

293FT (Thermo Fisher Scientific, R70007)

### DMEM complete medium

DMEM, high Glucose, GlutaMAX, pyruvate (Thermo Fisher Scientific, 11-965-118)

10% FBS (R&D systems, S11150)

100 U/mL Penicillin-Streptomycin (Thermo Fisher Scientific, 15-140-122)

### hGSC isolation medium

F12K (Thermo Fisher Scientific, 21127030)

2 mg/mL Collagenase IV (Worthington Biochemical, LS004188)

(optional) 2 U/mL DNase I (Worthington Biochemical, LS002007)

### hGSC medium

66.7% DMEM, high glucose (Thermo Fisher Scientific, 11-965-118)

33.3% F12K (Thermo Fisher Scientific, 21127030)

18% FBS (R&D systems, S11150)

10% R-Spondin-2 conditioned medium (RS2 cell line is a gift from Dr. Xi He from Children’s Hospital Boston; may be substituted by Recombinant Human R-Spondin 2 (PeproTech, 120-43))

10 mM Nicotinamide (Sigma-Aldrich, N5535)

25 μM Primocin (Invivogen, ant-pm-1)

1 μM A8301 (Cayman, 9001799)

5 μg/mL Insulin (Sigma-Aldrich, I0516-5ML)

10 μM Y-27632 (LC Laboratories, Y-5301)

1 μM DMH1 (Cayman, 16679)

50 ng/mL EGF (R&D Systems, 236-EG-01M)

2 μM T3 (Sigma-Aldrich, T6397)

### GINS medium

Advanced DMEM/F12 (Thermo Fisher Scientific, 12634010)

10 mM HEPES (Thermo Fisher Scientific, 15-630-080)

1X GlutaMAX (Thermo Fisher Scientific, 35050061)

1X B-27 (Themo Fisher Scientific, 17504044)

1X N-2 (Thermo Fisher Scientific, 17502048)

500 μM N-Acetyl-L-Cysteine (NAC) (Sigma-Aldrich, A9165)

25 μM Primocin (Invivogen, ant-pm-1)

10 mM Nicotinamide (Sigma-Aldrich, N5535)

1 μM A8301 (Cayman, 9001799)

10 μM Y-27632 (LC Laboratories, Y-5301)

## Equipment

Tissue culture humidified CO2 incubator

Biosafety cabinet

Water bath

P10 micropipette and sterile tips

P20 micropipette and sterile tips

P200 micropipette and sterile tips

P1000 micropipette and sterile tips

Pipet-Aid

5 mL sterile serological pipets (VWR, 89130-896)

10 mL sterile serological pipets (VWR, 89130-898)

25 mL sterile serological pipets (VWR, 89130-900)

15 mL centrifuge tube (VWR, 10026-076)

50 mL centrifuge tube (VWR, 10026-078)

Plate and tube centrifuge (Eppendorf, 5810R)

Aggrewell^™^ 400, 24 wells, Starter Kit (STEMCELL Technologies, 34450)

TC treated 48-well cell culture plate (VWR, 10062-898)

TC treated 6-well cell culture plate (VWR, 10062-892)

TC treated Dishes 100 × 20 mm (USA Scientific, CC7682-3394)

## Procedure

### Isolation of primary hGSCs from human stomach sample

1. Prepare 10 mL of hGSC isolation medium per stomach tissue sample (~2 cm^3^).
2. Warm the isolation medium at 37°C in water bath.
3. (**CRITICAL)** Cut tissue into tiny pieces that can go through 10-mL pipet.
4. Coat inner surface of a 10-mL pipet and a P1000 tip with BSA (1 % in PBS).
5. Wash tissue with 10 mL of cold DPBS with pipetting using the coated pipet.
6. Let the tissue sink to the bottom.
7. Discard supernatant.
8. Repeat step 5-7 until supernatant looks clean.
9. Spin down at 100 g for 3 min.
10. Discard supernatant.
11. Resuspend pellet in the warmed isolation medium using the coated pipet.
12. (**CRITICAL)** Incubate tissue at 37°C in the water bath for 15-30 min with pipetting rigorously using the coated 10 mL pipet (when the size of tissue allows, use the coated P1000 tip instead) until clusters of crypt cells released. NOTE: duration of this step should be adjusted case by case.
13. (Optional) Let stand for 1 min and collect the tissue that sink to the bottom and repeat step 11-12 in a separate tube.
14. Neutralize cells with F12K supplemented with 10% FBS.
15. Spin down at 500 g for 5 min.
16. Discard supernatant.
17. Resuspend pellet in hGSC medium and seed cells on 1 well of 6-well plate that coated with confluent inactivated MEF feeder cells.
18. Maintain cells in hGSC medium at 37 °C in a 5-7.5% CO_2_ humidified incubator.
19. Change medium every other day.

### Expansion and cryopreservation of hGSCs

**Note:** hGSC colonies should be passaged when the colonies begin to make contact to each other (~70% confluency).

1. Wash cells twice with DPBS.
2. Incubate cells in TrypLE for 10-12 min.
3. Detach cells by pipetting.
4. Transfer cells into a centrifuge tube that contains DMEM complete medium.
5. Centrifuge cells at 300 x g for 5 min.
6. Resuspend pellet in hGSC medium and then seed cells on an inactivated-MEF-coated dish. **Note:** Split hGSCs every 4-6 days at a ratio between 1:3 and 1:5.
7. Maintain hGSCs in hGSC medium at 37 °C in a 5-7.5% CO_2_ humidified incubator.
8. Change medium every other day.
9. For hGSC cryopreservation, pellet from step 6 should be resuspended in freezing solution (10% DMSO in FBS) and frozen using standard mammalian cell cryopreservation protocol.

### Generation of *Ngn3ER*-hGSC line

**Note:** *Ngn3ER*-hGSCs were labeled with mCherry constitutively by incorporation of a polycistronic cassette EF1a-*Ngn3ER-PuroR-mCherry* (*PuroR*, puromycin resistant gene).

1. Seed 10^5^ hGSCs in 1 well of 6-well plate coated with confluent inactivated MEF 24 hours prior to lentiviral transduction.
2. Wash cells with DPBS once.
3. Overlay cells with 2 mL of hGSC medium containing 10 μg/mL polybrene and ~10^6^ TU of Lenti-EF1a-*Ngn3ER-PuroR-mCherry*. **Note:** Lentivirus can be prepared in bulk and titrated in 293FT by mCherry^+^ cells quantification.
4. Spin the cell culture with lentivirus in plate at 1000 g for 30 min at 37°C.
5. Culture the infected hGSCs at 37°C in a 5-7.5% CO_2_ incubator for 48 hours.
6. Change culture medium to hGSC medium containing 1 μg/mL puromycin bidaily for 2 weeks.
7. The line can be expanded or cryopreserved.

### Generation of GINS organoids

#### 1. Ngn3ER activation (Differentiation to endocrine progenitors, day 0-2)

1. Seed *Ngn3ER*-hGSCs 4-5 days prior to differentiation.
2. Wash cells once with DPBS.
3. Overlay cells with hGSC medium containing 1 μM 4-OH-TAM.

#### 2. Pdx1-Mafa transduction (Differentiation to GINS precursors, day 2-6)

**Note:** coat the inner surface of pipet tips and centrifuge tubes with 1 % BSA before experiment.

1. Coat dishes in DMEM containing Fibronectin (1:50) and Matrigel (1:50) for 2 hours or overnight.
2. Gently wash cells with DPBS twice.
3. Incubate cells in DPBS for 10 min.*
4. Detach cells by pipetting vigorously using BSA-coated pipet tips. *
5. Transfer the cells in a BSA-coated centrifuge tube. *
6. Centrifuge at 100 g for 3 min.*
7. Remove supernatant carefully. * **Note**: cell debris should be removed if they retain in supernatant.
8. Dissociate cells in TrypLE at 37°C for 10-15 min with pipetting every 3-5 min.
9. (**CRITICAL)** Neutralize cells in complete DMEM when most of the cells are single cells, doublets or small clusters that contain 3-5 cells.
10. Spin cells at 100 g for 5 min.
11. Resuspend cells in medium composed of 50% of hGSC medium, 50% of GINS medium, and 10 μg/mL polybrene.
12. Infect cells with Lenti-CMV-*Pdx1-2A-Mafa* at a multiplicity of infection (MOI) of 10. **Note**: Lentivirus should be prepared in bulk and titrated in 293FT by immunostaining for Mafa and quantification.
13. Spin the cell culture with lentivirus in plate at 1000 x g for 30 min at 37°C.
14. Transfer infected cells to Fibronectin/Matrigel coated dishes (~10^7^ cells per 10-cm dish).
15. Two days post infection, change medium to 75% GINS medium and 25% hGSC medium.

*Note: for extremely large-scale production, consider skipping step 3-7, which was designed to remove sticky cell debris and undifferentiated hGSCs.

#### 3. GINS organoid formation (day 6-211)

1. Dissociate cells in TrypLE for 5 min.
2. Neutralize cells in DMEM complete medium.
3. Spin cells at 300 g for 5 min.
4. Resuspend pellet in GINS medium and count cells.
5. Aggregate cells in AggreWell400 (typically 2.0-2.4 million cells/well) using the manufacturer’s recommended protocol. **Note:** Aggregates normally form within 24 hours.
6. Change medium every 2-3 days.
7. Maintain organoids in GINS medium at 37 °C in a 5-7.5% CO_2_ humidified incubator.

## Troubleshooting

### Isolation of primary hGSCs from human stomach sample

**Step:** 3.

**Problem:** Tissue cannot go through 10-mL pipet.

**Possible reason:** Tissue is not cut thoroughly.

**Solution:** Put the tissue at the bottom of a sterile Eppendorf tube and use fine scissors to cut vigorously.

**Step:** 5.

**Problem:** Tissue stuck in pipets or tips.

**Possible reason:** The pipets/tips are not coated with BSA.

**Solution:** Make sure the pipets/tips are coated by pipetting the 1 *%* BSA solution a few times.

**Step:** 12.

**Problem:** No cell clusters release.

**Possible reason:** Did not use the right collagenase or did not pipet vigorously.

**Solution:** Make sure you use the right type of collagenase at the recommended concentration. After a few times of pipetting using 10-mL pipet, use BSA-coated P1000 tip to pipet.

### Expansion and cryopreservation of hGSCs

**Step:** 3.

**Problem:** Cells cannot detach.

**Possible reason:** (1) Cells have been neutralized before pipetting. (2) Colonies are too big and hard to detach.

**Solution:** (1) Detach the cells in TrypLE before neutralization. (2) Incubate cells in TrypLE for additional 2-3 min.

**Problem:** Colonies do not form in 3-4 days after passage.

**Possible reason:** Over-digestion.

**Solution:** Cells should remain in small clusters during the procedure. Single cells grow slowly.

### Generation of *Ngn3ER*-hGSC line

**Step:** 7.

**Problem:** No cells survive after selection.

**Possible reason:** The infection fails

**Solution:** Make sure the virus prep works.

**Problem:** Few cells are mCherry^+^ after selection.

**Possible reason:** Selection fails

**Solution:** Make sure the antibiotics can kill negative cells in 3-5 days. The concentration of the antibiotics may need optimization.

### Generation of GINS organoids

1. Ngn3ER activation (Differentiation to endocrine progenitors, day 0-2)

**Problem:** Morphology does not change on day 2

**Possible reason:** The morphological change on day 2 is subtle.

**Solution:** The morphology changes is cell line dependent. Some lines show more dramatic changes than the others. Clearer boundaries between cells in colonies should be observed.

2. Pdx1-Mafa transduction (Differentiation to GINS precursors, day 2-6)

**Step:** 4

**Problem:** Cells do not detach.

**Possible reason:** Cells are not differentiating.

**Solution:** Make sure the selection of *Ngn3ER*-hGSC works and the cells can differentiate. Without Pdx1-Mafa transduction, cells should differentiate into gastric endocrine cells.

3. GINS organoid formation (day 6-21)

**Problem:** Endocrine progenitors do not differentiate into GINS cells

**Possible reason:** Lentivirus transduction fails

**Solution:** Make sure the virus prep works; MOI may need optimization; dissociate the endocrine progenitors into single cells or doublets to increase infectivity; polybrene should be added during infection; use immunostaining to confirm the expression of Pdx1/Mafa.

## Time Taken

### Isolation of primary hGSCs from human stomach sample

2-3 hours

### Expansion and cryopreservation of hGSCs

1 hour based on one 10-cm dish scale.

### Generation of *Ngn3ER*-hGSC line

1.5 hours excluding antibiotics selection

### Generation of GINS organoids

1. Ngn3ER activation (Differentiation to endocrine progenitors, day 0-2)

5 min

2. Pdx1-Mafa transduction (Differentiation to GINS precursors, day 2-6)

2 hours

3. GINS organoid formation (day 6-21)

1 hour

## Anticipated Results

### Isolation of primary hGSCs from human stomach sample

30-40 primary hGSC colonies would be visible in 1-2 weeks.

### Expansion and cryopreservation of hGSCs

Cell doubling time is approximately 48 hours.

Mucus may be secreted by spontaneously differentiated cells.

### Generation of *Ngn3ER*-hGSC line

Approximately 99% of the cells should be mCherry^+^ after the procedure.

### Generation of GINS organoids

1. Ngn3ER activation (Differentiation to endocrine progenitors, day 0-2):

Colonies begin to fall apart. Cell morphology changes. Cells stop growing. Some cells die.

2. Pdx1-Mafa transduction (Differentiation to GINS precursors, day 2-6):

Cells spontaneously cluster on day 5-6. Some cells die.

3. GINS organoid formation (day 6-21):

Cells aggregates into organoid within 24 hours. Organoids secret insulin in response to glucose as early as 4 days post aggregation.

## References

[1] Gehart H. & Clevers H. Tales from the crypt: new insights into intestinal stem cells. Nat Rev Gastroenterol Hepatol 16, 19–34, doi:10.1038/s41575-018-0081-y (2019). 30429586

[2] Wells J. M. & Spence J. R. How to make an intestine. Development 141, 752–760, doi:10.1242/dev.097386 (2014). 24496613 PMC3912826

[3] Santos A. J. M., Lo Y. H., Mah A. T. & Kuo C. J. The Intestinal Stem Cell Niche: Homeostasis and Adaptations. Trends Cell Biol 28, 1062–1078, doi:10.1016/j.tcb.2018.08.001 (2018). 30195922 PMC6338454

[4] Sugimoto S. An organoid-based organ-repurposing approach to treat short bowel syndrome. Nature 592, 99–104, doi:10.1038/s41586-021-03247-2 (2021). 33627870

[5] Nikolaev M. Homeostatic mini-intestines through scaffold-guided organoid morphogenesis. Nature 585, 574–578, doi:10.1038/s41586-020-2724-8 (2020). 32939089

[6] Meran L. Engineering transplantable jejunal mucosal grafts using patient-derived organoids from children with intestinal failure. Nat Med 26, 1593–1601, doi:10.1038/s41591-020-1024-z (2020). 32895569 PMC7116539

[7] Egozi A. Insulin is expressed by enteroendocrine cells during human fetal development. Nat Med 27, 2104–2107, doi:10.1038/s41591-021-01586-1 (2021). 34887578

[8] Warshauer J. T., Bluestone J. A. & Anderson M. S. New Frontiers in the Treatment of Type 1 Diabetes. Cell Metab 31, 46–61, doi:10.1016/j.cmet.2019.11.017 (2020). 31839487 PMC6986815

[9] Zhou Q. & Melton D. A. Pancreas regeneration. Nature 557, 351–358, doi:10.1038/s41586-018-0088-0 (2018). 29769672 PMC6168194

[10] Brusko T. M., Russ H. A. & Stabler C. L. Strategies for durable beta cell replacement in type 1 diabetes. Science 373, 516–522, doi:10.1126/science.abh1657 (2021). 34326233 PMC8867839

[11] Ramzy A. Implanted pluripotent stem-cell-derived pancreatic endoderm cells secrete glucose-responsive C-peptide in patients with type 1 diabetes. Cell Stem Cell 28, 2047–2061 e2045, doi:10.1016/j.stem.2021.10.003 (2021). 34861146

[12] Sneddon J. B. Stem Cell Therapies for Treating Diabetes: Progress and Remaining Challenges. Cell Stem Cell 22, 810–823, doi:10.1016/j.stem.2018.05.016 (2018). 29859172 PMC6007036

[13] Millman J. R. & Pagliuca F. W. Autologous Pluripotent Stem Cell-Derived beta-Like Cells for Diabetes Cellular Therapy. Diabetes 66, 1111–1120, doi:10.2337/db16-1406 (2017). 28507211

[14] Ariyachet C. Reprogrammed Stomach Tissue as a Renewable Source of Functional beta Cells for Blood Glucose Regulation. Cell Stem Cell 18, 410–421, doi:10.1016/j.stem.2016.01.003 (2016). 26908146 PMC4779391

[15] Gu W., Colarusso J. L., Dame M. K., Spence J. R. & Zhou Q. Rapid establishment of human colonic organoid knockout lines. STAR Protoc 3, 101308, doi:10.1016/j.xpro.2022.101308 (2022). 35463469 PMC9026586

[16] Gu W. SATB2 preserves colon stem cell identity and mediates ileum-colon conversion via enhancer remodeling. Cell Stem Cell, doi:10.1016/j.stem.2021.09.004 (2021). PMC8741647 34582804

